# Esophageal Perforation with Unilateral Fluidothorax Caused by Nasogastric Tube

**DOI:** 10.1155/2016/4103734

**Published:** 2016-10-10

**Authors:** Lukas P. Mileder, Martin Müller, Friedrich Reiterer, Alexander Pilhatsch, Barbara Gürtl-Lackner, Berndt Urlesberger, Wolfgang Raith

**Affiliations:** ^1^Division of Neonatology, Department of Pediatrics and Adolescent Medicine, Medical University of Graz, Graz, Austria; ^2^Division of Pediatric Radiology, Department of Radiology, Medical University of Graz, Graz, Austria; ^3^Institute of Pathology, Medical University of Graz, Graz, Austria; ^4^Department of Pathology, Skåne Regional and University Laboratories, Lund, Sweden

## Abstract

Preterm infants are highly susceptible to injuries following necessary and often life-saving medical interventions. Esophageal perforation is a rare, yet serious complication that can be caused by aerodigestive tract suction, endotracheal intubation, or nasogastric tube placement. We present the case of a neonate born at 23 weeks plus three days of gestation with chest radiography showing malposition of the nasogastric feeding tube and massive right-sided effusion of Iopamidol in the pleural cavity due to esophageal perforation. In addition, the article summarizes common signs and symptoms associated with esophageal perforation in infants and discusses diagnostic approaches.

## 1. Introduction

Esophageal perforation (EP) is a rare, yet serious complication [1], with an incidence of 1 : 124 among preterm infants weighing less than 1500 g at birth [2]. Mortality is as high as 20% in these vulnerable patients [3].

## 2. Case Presentation

A female twin neonate born at 23 weeks plus three days of gestation with a birth weight of 538 g was intubated after birth due to respiratory distress and received surfactant repeatedly. Because of signs of evolving bronchopulmonary dysplasia and pulmonary hypertension, she was treated with hydrocortisone and nitric oxide. Transcranial sonography revealed grade II intraventricular hemorrhage.

**Figure 1:**
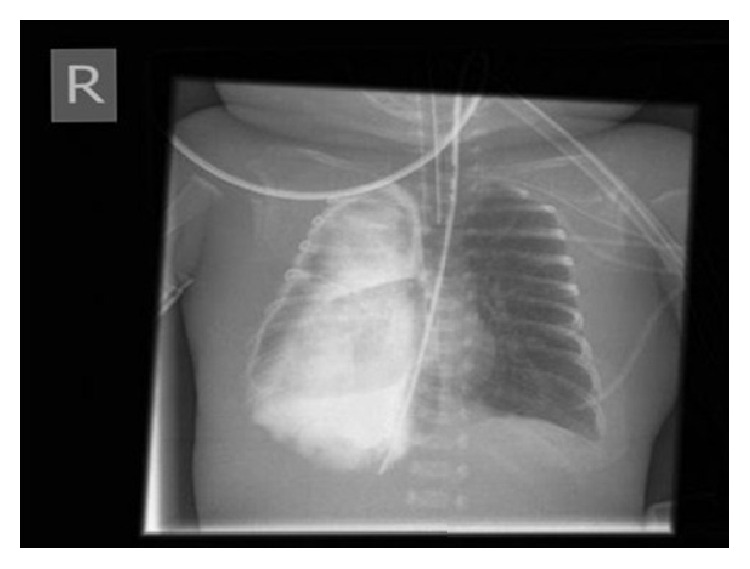
Chest radiograph after contrast agent application showing massive effusion in the pleural cavity and abnormal right-sided position of the nasogastric tube.

On day 8 of life, feeding problems with bilious gastric residuals, constipation, abdominal distension, and lactic acidosis developed, which were initially managed conservatively. Spontaneous ileum perforation necessitated laparoscopic surgery, including local bowel resection and creation of an ileostomy, on day 12. One day after surgery, clinical signs suggested development of an intestinal volvulus. After injection of Iopamidol for evaluation of intestinal obstruction, chest radiography showed massive right-sided effusion in the pleural cavity and malposition of the nasogastric feeding tube (Figure 1). Pleural drainage resulted in prompt stabilization of vital parameters after extraction of 10 mL of serous liquid. Nitric oxide therapy was gradually reduced and could be abandoned on day 13.

Due to numerous morbidities including intraventricular hemorrhage and arterial hypotension despite inotropic support, the neonate died on day 20. Autopsy showed regular esophageal development with minimal inflammatory reaction below the muscular layer and found lung immaturity to be the cause of death.

## 3. Discussion

Iatrogenic EP can be caused by vigorous pharyngoesophageal suction, traumatic endotracheal intubation, and insertion of naso-/orogastric feeding tubes [4]. Clinical signs and symptoms of EP include inability to pass a gastric tube, feeding difficulties, bloody oral secretions, fever, cyanosis, and subcutaneous emphysema [4, 5]. In our case, EP did not present with sudden respiratory deterioration, which is the most common symptom [1]. Apparently, there is a wide spectrum of the clinical presentation of EP especially in extremely preterm infants, including malposition of the nasogastric feeding tube.

Diagnosis of EP is based on clinical findings, chest radiography, and endoscopy [3]. Radiographic evidence of EP includes pneumothorax, pneumomediastinum, or subcutaneous air [4], with proximal EP usually causing left-sided abnormalities and distal perforations commonly appearing on the right side [6]. Flexible endoscopy has been shown to allow for diagnosis and precise location of EP at the bedside while avoiding contrast use and radiation exposure [7] and should therefore be considered as diagnostic measure especially in preterm infants with clinical signs of EP.
